# Impact of everolimus plus calcineurin inhibitor on formation of non-HLA antibodies and graft outcomes in kidney transplant recipients: 12-month results from the ATHENA substudy

**DOI:** 10.3389/frtra.2023.1273890

**Published:** 2023-11-21

**Authors:** Aurélie Philippe, Wolfgang Arns, Vanessa Ditt, Ingeborg A. Hauser, Friedrich Thaiss, Claudia Sommerer, Barbara Suwelack, Duska Dragun, Jan Hillen, Christiane Schiedel, Anja Elsässer, Björn Nashan

**Affiliations:** ^1^ BIH Biomedical Innovation Academy, Berlin Institute of Health at Charité—Universitätsmedizin Berlin, Berlin, Germany; ^2^ Charité—Universitätsmedizin Berlin, Corporate Member of Freie Universität Berlin and Humboldt-Universität zu Berlin, Clinic for Nephrology and Critical Care Medicine, Campus Virchow-Klinikum, Berlin, Germany; ^3^ Transplant Centre Cologne, Cologne General Hospital, Cologne, Germany; ^4^ Institute of Transfusion Medicine, Kliniken der Stadt Köln, Cologne, Germany; ^5^ Department of Nephrology, University Hospital Frankfurt, Goethe University Frankfurt, Frankfurt, Germany; ^6^ III. Department of Medicine, University Medical Center Hamburg-Eppendorf, Hamburg, Germany; ^7^ Nephrology, Kidney Center Heidelberg, University Hospital Heidelberg, Heidelberg, Germany; ^8^ Department of Internal Medicine, Transplant Nephrology, University Hospital of Münster, Münster, Germany; ^9^ Immunology, Novartis Pharma GmbH, Nürnberg, Germany; ^10^ Department of Hepatobiliary Surgery and Visceral Transplantation, University Medical Center Hamburg-Eppendorf, Hamburg, Germany; ^11^ Organ Transplantation Center, The First Affiliated Hospital of University of Science and Technology of China, Anhui Provincial Hospital, Hefei, China

**Keywords:** non-human leukocyte antigen, everolimus, kidney transplantation, angiotensin II type 1 receptor, endothelin-1 type A receptor

## Abstract

**Background:**

Non-human leukocyte antigen (non-HLA) antibodies including antibodies targeting Angiotensin II type 1 (AT1R) and Endothelin-1 type A (ETAR) receptors represent a topic of interest in kidney transplantation (KTx). This exploratory substudy evaluated the impact of everolimus (EVR) or mycophenolic acid (MPA) in combination with tacrolimus (TAC) or cyclosporine A (CsA) in patients with preformed non-HLA antibodies, potentially associated rejections and/or their impact on renal function over 1 year.

**Methods:**

All eligible patients were randomized (1:1:1) before transplantation to receive either EVR/TAC, EVR/CsA, or MPA/TAC regimen. The effect of these regimens on the formation of non-HLA antibodies within one year post *de novo* KTx and the association with clinical events was evaluated descriptively in randomized (*n* = 268) population.

**Results:**

At Month 12, in EVR/TAC group, higher incidence of patients negative for AT1R- and ETAR-antibodies (82.2% and 76.7%, respectively) was noted, whereas the incidence of AT1R- and ETAR-antibodies positivity (28.1% and 34.7%, respectively) was higher in the MPA/TAC group. Non-HLA antibodies had no influence on clinical outcomes in any treatment group and no graft loss or death was reported.

**Conclusions:**

The studied combinations of immunosuppressants were safe with no influence on clinical outcomes and suggested minimal exposure of calcineurin inhibitors for better patient management.

**Clinical Trial Registration:**

https://clinicaltrials.gov/ (NCT01843348; EudraCT number: 2011-005238-21).

## 1. Introduction

Non-human leukocyte antigen (non-HLA) antibodies (Abs) against Angiotensin II type 1 receptor Abs (AT1R-Abs) and Endothelin-1 type A receptor Abs (ETAR-Abs) are responsible for activating signaling pathways associated with cell proliferation, vascular injury (1), increased risk of allograft rejection (2–5) and fibrosis in solid organ transplant recipients (6, 7) implicating their impact on post-transplant morbidity and mortality. Banasik et al. revealed that the existence of ETAR-Abs is linked to decreased kidney transplant (KTx) function in the first 12 months after transplantation (8). In KTx patients, high levels of AT1R- and/or ETAR-Abs are associated with morphological and functional allograft injury and graft loss (8–11). Further, increased levels of AT1R- and ETAR-Abs are also linked to cellular and antibody-mediated rejection with an influence on the early onset of microvasculopathy in patients after heart transplantation (12). Sorohan et al. reported that immunosuppression (induction and maintenance) with anti-thymocyte globulin and immediate-release tacrolimus (TAC) are independent risk factors for the development of non-HLA antibodies in KTx recipients (13). Considering the important role of non-HLA Abs on graft outcomes (14–16) evaluating the impact of immunosuppression on these Abs and clinical outcomes after organ transplantation is still an unmet need.

Everolimus (EVR), a mammalian target of rapamycin inhibitor (mTORi), acts through mechanisms complementary to calcineurin inhibitors (CNIs), such as TAC, thereby allowing reduction of CNI exposure (17). In addition, EVR is also associated with a decreased incidence of cancer (18, 19), and a beneficial effect on cardiovascular stability (20). Moreover, studies have reported that EVR reduces infections (21), particularly CMV (22–26) and BKV infections (26, 27), and has shown to improve COVID-19 vaccination response in KTx recipients (28). In a phase 3b, multicenter, open-label, 24-month study of 833 *de novo* (who received a first kidney transplant) living or deceased donor KTx recipients, the use of EVR was associated with a more than 60% reduction in cyclosporine A (CsA) exposure with comparable efficacy and renal function to a mycophenolic acid plus standard-exposure CsA (MPA + sCsA) regimen over a 2-year period (17, 29).

ATHENA, the to date largest randomized European kidney transplantation study, was a 12-month, prospective, multicenter, randomized, parallel group, open-label study undertaken in 612 *de novo* KTx recipients (ClinicalTrials.gov identifier: NCT01843348; EudraCT number: 2011-005238-21) that compared EVR in combination with TAC (EVR + TAC) or CsA (EVR + CsA) vs. MPA + TAC. The 12-month results from the ATHENA study showed that EVR + TAC or CsA has comparable efficacy to MPA + TAC although non-inferiority of renal function with EVR + TAC/CsA was not achieved (26). Given the paucity of literature on the effect of the immunosuppression on non-HLA antibody levels and their effects in *de novo* KTx recipients, this exploratory substudy in patients from the ATHENA study was the first to evaluate the impact of EVR or MPA in combination with CNI on preformed non-HLA antibody, potentially associated rejections and/or its impact on renal function from the time of transplantation (baseline) to one year.

## 2. Materials and methods

### 2.1. Study design and conduct

ATHENA was a 12-month, prospective, multicenter, randomized, controlled, parallel group, open label study in *de novo* KTx recipients. The study was conducted from December 27, 2012, through March 23, 2016, in Germany and France. Methods for the ATHENA study, including inclusion/exclusion criteria, the immunosuppression regimen, and patient stratification have been described in detail previously (26). Briefly, all eligible patients of low to moderate immunological risk were randomized in a 1:1:1 ratio prior to transplantation to receive either EVR/TAC, EVR/CsA, or MPA/TAC regimen (26, 30). All patients received basiliximab 20-mg induction therapy on day 0 and 4, and a minimum dose of 5-mg prednisolone or equivalent until month 12 (M12). The target EVR trough concentration in the EVR/TAC and EVR/CsA groups was in the range of 3 to 8 ng/ml throughout the study period. In the EVR/TAC and MPA/TAC groups, the target TAC trough concentration was in the range of 4 to 8 ng/ml until the end of M2 and in the range of 3 to 5 ng/ml thereafter. The CsA target range in the EVR/CsA group was 75 to 125 ng/ml until the end of M2 and 50 to 100 ng/ml thereafter. In the MPA/TAC group, MPA was given as enteric-coated mycophenolate sodium (1.44 g/d) or mycophenolate mofetil (2 g/d).

The study protocol and all amendments were reviewed and approved by the independent ethics committee or institutional review board for each center. The study was conducted according to the ethical principles of the Declaration of Helsinki. Informed consent was obtained from each patient in writing before randomization.

### 2.2. Study outcomes

This substudy investigated the effect of the three immunosuppressive regimens on preformednon-HLA Abs, potentially associated rejections and/or its impact on renal function over one year post *de novo* KTx. Blood samples of 5 ml were collected for all patients on the day of transplantation (baseline), M6 and M12. AT1R- and ETAR-Abs were measured by ELISA assays using commercially available kits (CellTrend GmbH, Luckenwalde, Germany) according to manufacturer's instructions. The samples were assayed on ELISA plates which were pre-coated with membrane extracts from Chinese Hamster Ovary cells overexpressing the human receptors AT1R and ETAR individually in their native conformation. Plates were then incubated for two hours at 4°C with serum samples obtained from patients. Following washing, plates were incubated with a labeled secondary antibody detecting human Abs. After a second phase of washing, presence of the labeled antibody was revealed, and optical density measured in Units/ml (U/ml) represented the level of non-HLA Abs in the serum samples (8).

### 2.3. Statistical analysis

Patients who received at least one dose of the study drug were considered in the intent-to-treat (ITT) population, and all ITT patients without any major protocol deviation were considered in the per-protocol (PP) population. The analysis set included all transplanted patients with at least one antibody assessment (either HLA and/or non-HLA). The analysis of non-HLA Abs was carried out descriptively. The embedded subgroups of non-HLA Abs were defined as follows: preformed non-HLA AT1R-Abs: >10 U/ml at baseline; preformed non-HLA ETAR-Abs: >10 U/ml at baseline.

## 3. Results

A total of 612 patients were included in this study. Since the non-HLA Abs data analysis was comparable in ITT (*n* = 478/612) and PP (*n* = 268/612), here we reported only the PP results (*n* = 268; EVR/TAC *n* = 91, EVR/CsA *n* = 57 and MPA/TAC *n* = 120 pts) (Table 1). The baseline and demographic details of the patients are presented in Table 2 and Supplementary Table S1. At baseline, 136 (51%) showed positivity for ETAR-Abs and 118 (44%) showed positivity for AT1R-Abs (Table 1); of which 34 (12.7%) patients showed positivity for only ETAR-Abs, 16 (6.0%) patients for only AT1R-Abs, whereas 102 patients (38.1%) showed positivity for both ETAR- and AT1R-Abs and 116 patients (43.3%) showed absence of both non-HLA Abs (cutoff >10 U/ml) (Supplementary Table S1). At baseline, higher incidence of patients positive for only AT1R- and only ETAR-Abs (42.9% and 51.6%, respectively) were noted in the EVR/CsA group, whereas in MPA/TAC, 40.8% and 46.7% patients were positive for only AT1R- and only ETAR-Abs, respectively (Table 3). At M6, no difference was noted in patients positive and negative for only AT1R-Abs in all treatment groups, whereas higher incidence of patients positive for ETAR-Abs were noted in EVR/CsA group and no difference was observed in patients negative for ETAR-Abs. At M12, the incidence of only AT1R- and only ETAR-Abs positivity (28.1% and 34.7%, respectively) was higher in the MPA/TAC group, whereas the higher incidence of patients negative for only AT1R- and only ETAR-Abs (82.2% and 76.7%, respectively) was noted in the EVR/TAC group. The number of patients with positive AT1R- and ETAR-Abs decreased at M6 in all treatment groups and at M12 in the EVR/TAC group while it increased slightly in MPA/TAC group at M12 (Table 3).

**Table 1:** Number of patients in ATHENA substudy cohort with non-HLA antibody data.

Patient group	Non-HLA data at BL	Preformed AT1R-Abs	Preformed AT1R-Abs + clinical outcome	Preformed ETAR-Abs	Preformed ETAR-Abs + clinical outcome
	*N*	*N* (%)	*N* (%)	*N* (%)	*N* (%)
ITT					
EVR/TAC	164	73 (44.5)	11 (15.1)	83 (50.6)	13 (15.7)
EVR/CsA	149	77 (51.7)	21 (27.3)	81 (54.4)	18 (22.2)
MPA/TAC	165	68 (41.2)	7 (10.3)	77 (46.7)	7 (9.1)
PP					
EVR/TAC	91	39 (42.9)	1 (2.6)	47 (51.6)	1 (2.1)
EVR/CsA	57	30 (52.6)	3 (10.0)	33 (57.9)	3 (9.1)
MPA/TAC	120	49 (40.8)	2 (4.1)	56 (46.7)	1 (1.8)

Preformed non-HLA AT1R-Abs: >10 U/ml at baseline; preformed non-HLA ETAR-Abs: >10 U/ml at baseline.

Abs, antibodies; AT1R, angiotensin II type 1 receptor; BL, baseline; CsA, cyclosporine; eGFR, estimated glomerular filtration rate; EVR, everolimus; ETAR, endothelin-1 type A receptor; HLA, human leukocyte antigen; ITT, intention to treat; MPA, mycophenolic acid; PP, per-protocol; TAC, tacrolimus.

**Table 2:** Baseline and demographic details of patients with non-HLA antibodies.

Variable	Negative non-HLA ETAR-Abs	Positive non-HLA ETAR-Abs	Total	*p*- value	Negative non-HLA AT1R-Abs	Positive non-HLA AT1R-Abs	Total	*p*- value
	(*N* = 132)	(*N* = 136)	(*N* = 268)		(*N* = 150)	(*N* = 118)	(*N* = 268)	
	*n* (%)	*n* (%)	*n* (%)		*n* (%)	*n* (%)	*n* (%)	
Age (yrs)								
*n*; Mean (SD)	132; 51.1 (11.5)	136; 55.2 (11.7)	268; 53.1 (11.7)	0.004^a^	150; 52.3 (11.6)	118; 54.2 (11.9)	268; 53.1 (11.7)	0.201^a^
Age, *n* (%)								
<65 yrs	115 (87.1)	109 (80.1)	224 (83.6)		131 (87.3)	93 (78.8)	224 (83.6)	
≥65 yrs	17 (12.9)	27 (19.9)	44 (16.4)		19 (12.7)	25 (21.2)	44 (16.4)	
Gender, *n* (%)								
Male	86 (65.2)	96 (70.6)	182 (67.9)	0.362^b^	102 (68.0)	80 (67.8)	182 (67.9)	1.000^b^
Female	46 (34.8)	40 (29.4)	86 (32.1)		48 (32.0)	38 (32.2)	86 (32.1)	
Race, *n* (%)								
Caucasian	127 (96.2)	133 (97.8)	260 (97.0)	0.496^b^	146 (97.3)	114 (96.6)	260 (97.0)	0.734^b^
Non-Caucasian	5 (3.8)	3 (2.2)	8 (3.0)		4 (2.7)	4 (3.4)	8 (3.0)	* *
Body Mass Index								
*n*; Mean (SD)	132; 26.1 (4.3)	136; 26.3 (3.8)	268; 26.2 (4.0)	0.800^a^	150; 26.1 (4.1)	118; 26.4 (3.9)	268; 26.2 (4.0)	0.474^a^
Panel reactive antibodies, *n* (%)								
0	121 (95.3)	125 (96.9)	246 (96.1)		136 (96.5)	110 (95.7)	246 (96.1)	
≤10	5 (3.9)	3 (2.3)	8 (3.1)		4 (2.8)	4 (3.5)	8 (3.1)	
>10 and ≤20	1 (0.8)	0 (0.0)	1 (0.4)		1 (0.7)	0 (0.0)	1 (0.4)	
>20	0 (0.0)	1 (0.8)	1 (0.4)		0 (0.0)	1 (0.9)	1 (0.4)	
Missing	5	7	12		9	3	12	
(One or more) Previous kidney transplant, *n* (%)								
No	127 (96.2)	135 (99.3)	262 (97.8)		145 (96.7)	117 (99.2)	262 (97.8)	
Yes	5 (3.8)	1 (0.7)	6 (2.2)		5 (3.3)	1 (0.8)	6 (2.2)	
HLA-A mismatches, *n* (%)								
0	43 (32.6)	34 (25.0)	77 (28.7)		41 (27.3)	36 (30.5)	77 (28.7)	
1	65 (49.2)	66 (48.5)	131 (48.9)		75 (50.0)	56 (47.5)	131 (48.9)	
2	24 (18.2)	36 (26.5)	60 (22.4)		34 (22.7)	26 (22.0)	60 (22.4)	
HLA-B mismatches, *n* (%)								
0	38 (28.8)	22 (16.2)	60 (22.4)		33 (22.0)	27 (22.9)	60 (22.4)	
1	54 (40.9)	55 (40.4)	109 (40.7)		58 (38.7)	51 (43.2)	109 (40.7)	
2	40 (30.3)	59 (43.4)	99 (36.9)		59 (39.3)	40 (33.9)	99 (36.9)	
HLA-DR mismatches, *n* (%)								
0	47 (35.6)	38 (27.9)	85 (31.7)		47 (31.3)	38 (32.2)	85 (31.7)	
1	69 (52.3)	69 (50.7)	138 (51.5)		77 (51.3)	61 (51.7)	138 (51.5)	
2	16 (12.1)	29 (21.3)	45 (16.8)		26 (17.3)	19 (16.1)	45 (16.8)	
Cold ischemia time [h]								
*n*; Mean (SD)	132; 10.4 (6.0)	136; 11.6 (6.1)	268; 11.0 (6.0)		150; 10.9 (6.1)	118; 11.1 (5.9)	268; 11.0 (6.0)	
Donor age (yrs)								
*n*; Mean (SD)	132; 48.4 (15.5)	136; 54.4 (14.3)	268; 51.4 (15.2)		150; 49.8 (15.9)	118; 53.5 (14.0)	268; 51.4 (15.2)	
Kind of donation, *n* (%)								
Deceased heart beating	105 (79.5)	115 (84.6)	220 (82.1)		120 (80.0)	100 (84.7)	220 (82.1)	
Living related	18 (13.6)	12 (8.8)	30 (11.2)		19 (12.7)	11 (9.3)	30 (11.2)	
Living unrelated	9 (6.8)	9 (6.6)	18 (6.7)		11 (7.3)	7 (5.9)	18 (6.7)	

Abs, antibodies; AT1R, angiotensin II type 1 receptor; CsA, cyclosporine A; ETAR, endothelin-1 type A receptor; EVR, everolimus; h, hours; HLA, human leukocyte antigens; MPA, mycophenolic acid; SD, standard deviation; TAC, tacrolimus; yrs, years.

^a^ *t*-test.

^b^ Fisher's exact test. Preformed non-HLA AT1R-Abs: >10 U/ml at baseline; preformed non-HLA ETAR-Abs: >10 U/ml at baseline.

**Table 3:** Incidences of only AT1R-antibodies and only ETAR-antibodies at baseline, month 6 and month 12 by treatment (PP).

Variables	MPA + TAC	EVR + TAC	EVR + CsA
	(*N* = 147)	(*N* = 110)	(*N* = 80)
	*n* (%)	*n* (%)	*n* (%)
% of patients with only AT1R-Abs			
Patients with any non-HLA data	129 (87.8)	98 (89.1)	70 (87.5)
Baseline			
Negative	71/120 (59.2)	52/91 (57.1)	27/57 (47.4)
Positive	49/120 (40.8)	39/91 (42.9)	30/57 (52.6)
Missing	9	7	13
Month 6			
Negative	90/114 (78.9)	70/88 (79.5)	48/61 (78.7)
Positive	24/114 (21.1)	18/88 (20.5)	13/61 (21.3)
Missing	15	10	9
Month 12			
Negative	87/121 (71.9)	74/90 (82.2)	51/67 (76.1)
Positive	34/121 (28.1)	16/90 (17.7)	16/67 (23.9)
Missing	8	8	3
% of patients with only ETAR-Abs			
Patients with any non-HLA data	129 (87.8)	98 (89.1)	70 (87.5)
Baseline			
Negative	64/120 (53.3)	44/91 (48.4)	24/57 (42.1)
Positive	56/120 (46.7)	47/91 (51.6)	33/57 (57.9)
Missing	9	7	13
Month 6			
Negative	86/114 (75.4)	66/88 (75.0)	39/61 (63.9)
Positive	28/114 (24.6)	22/88 (25.0)	22/61 (36.1)
Missing	15	10	9
Month 12			
Negative	79/121 (65.3)	69/90 (76.7)	46/67 (68.7)
Positive	42/121 (34.7)	21/90 (23.3)	21/67 (31.3)
Missing	8	8	3

Preformed means AT1R-Abs >10 U/ml at baseline; preformed means ETAR-Abs >10 U/ml at baseline.

Abs, antibodies; AT1R, angiotensin II type 1 receptor; CsA, cyclosporine; ETAR, endothelin-1 type A receptor; EVR, everolimus; MPA, mycophenolic acid; PP, per-protocol; TAC, tacrolimus.

Preformed non-HLA Abs had no influence on clinical outcome in all treatment groups. At M12, out of 118 patients positive for only AT1R-Abs, only 1/39 (2.6%, EVR + TAC), 3/30 (10%, EVR + CsA) and 2/49 (4.1%, MPA + TAC) reported incidence of BPAR (Table 4). In patients positive for only ETAR-Abs (*N* = 136), similar figures were noted with 1/47 (2.1%, EVR + TAC), 3/33 (9.1%, EVR + CsA) and 1/56 (1.8%, MPA + TAC) patients who experienced BPAR at M12 (Table 4). Similarly in patients without non-HLA Abs, the number of BPAR events were very small in EVR/TAC and EVR/CsA groups but increased slightly in MPA/TAC group compared to patients with preformed non-HLA Abs. No graft loss or death was reported in any treatment groups irrespective of non-HLA Abs status at baseline.

**Table 4:** Association of non-HLA antibodies expression and clinical outcome at month 12 by treatment.

Patients with preformed^a^ non-HLA AT1R-Abs				Patients without preformed^a^ non-HLA AT1R-Abs		
Clinical outcomes	MPA + TAC	EVR + TAC	EVR + CsA	MPA + TAC	EVR + TAC	EVR + CsA
	(*N* = 49)	(*N* = 39)	(*N* = 30)	(*N* = 71)	(*N* = 52)	(*N* = 27)
	*n* (%)	*n* (%)	*n* (%)	*n* (%)	*n* (%)	*n* (%)
BPAR	2 (4.1)	1 (2.6)	3 (10.0)	5 (7.0)	1 (1.9)	1 (3.7)
Type IA	1 (2.0%)	1 (2.6)	3 (10.0)	1	1 (1.9)	–
Type IIB	0	0	0	1	0	–
Evidence of AMR	0	1 (2.6)	1 (3.3)	1 (1.4)	0	0
No evidence of AMR	2 (4.1)	0	2 (6.7)	4 (5.6)	1 (1.9)	1 (3.7)
eGFR change (ml/min/1.73 m^2^)	+10.1	+3.2	−2.7	+6.9	+3.8	+5.9
Graft loss	0	0	0	0	0	0
Death	0	0	0	0	0	0
Patients with preformed^b^ non-HLA ETAR-Abs				Patients without preformed^b^ non-HLA ETAR-Abs		
** **	MPA + TAC	EVR + TAC	EVR + CsA	MPA + TAC	EVR + TAC	EVR + CsA
	(*N* = 56)	(*N* = 47)	(*N* = 33)	(*N* = 64)	(*N* = 44)	(*N* = 24)
	*n* (%)	*n* (%)	*n* (%)	*n* (%)	*n* (%)	*n* (%)
BPAR	1 (1.8)	1 (2.1)	3 (9.1)	6 (9.4)	1 (2.3)	1 (4.2)
Type IA	1 (1.8)	1 (2.1)	2	1	1 (2.3)	1 (4.2)
Type IIB	0	0	0	1	0	0
Evidence of AMR	0	1 (2.1)	1 (3.0)	1 (1.6)	0	0
No evidence of AMR	1 (1.8)	0	2 (6.1)	5 (7.8)	1 (2.3)	1 (4.2)
eGFR change (ml/min/1.73 m^2^)	+7.0	+5.0	−1.5	+9.2	+2.1	+5.4
Graft loss	0	0	0	0	0	0
Death	0	0	0	0	0	0

Abs, antibodies; AT1R, angiotensin II type 1 receptor; BPAR, biopsy-proven acute rejection; CsA, cyclosporine A; EVR, everolimus; ETAR, endothelin-1 type A receptor; MPA, mycophenolic acid; TAC, tacrolimus.

^a^ Preformed means AT1R-Abs >10 U/ml at baseline.

^b^ Preformed means ETAR-Abs >10 U/ml at baseline.

**Figure 1:**
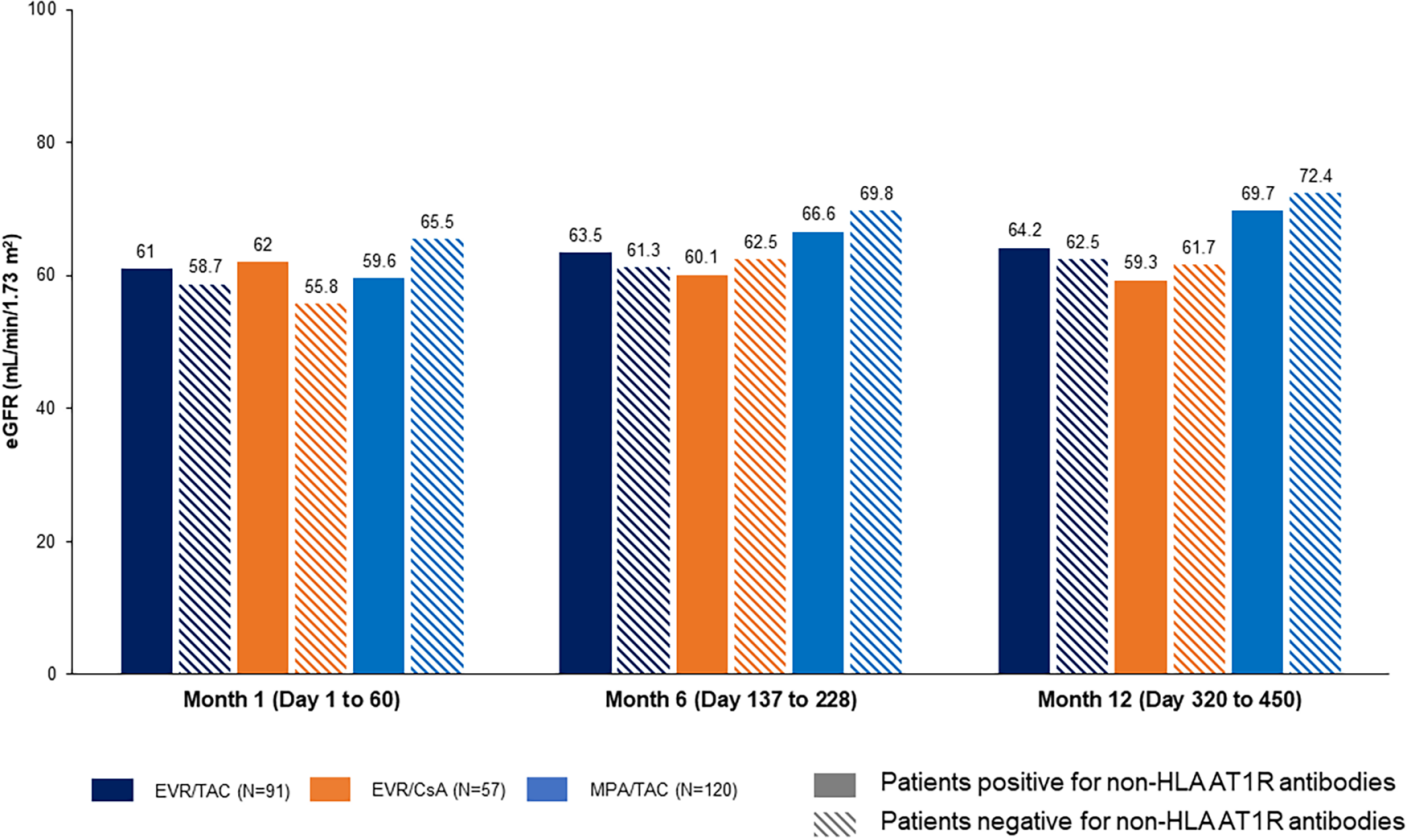
Influence of preformed non-HLA AT1R-antibodies on eGFR (Nankivell). Preformed non-HLA AT1R-Abs: >10 U/ml at baseline. Abs, antibodies; AT1R, angiotensin II type 1 receptor; CsA, cyclosporine; eGFR, estimated glomerular filtration rate; EVR, everolimus; HLA, human leukocyte antigens; MPA, mycophenolic acid; TAC, tacrolimus.

**Figure 2:**
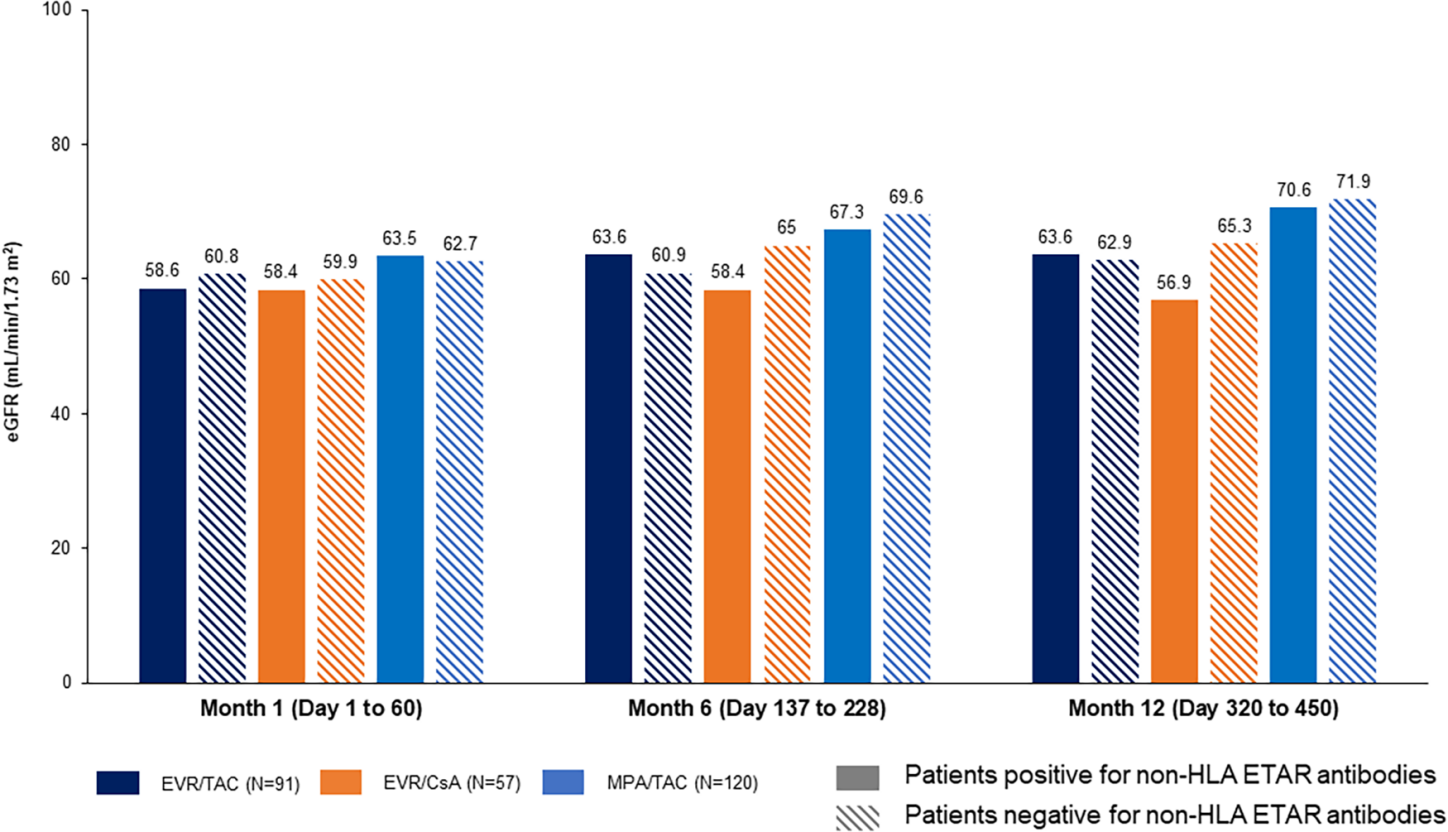
Influence of preformed non-HLA ETAR-antibodies on eGFR (Nankivell). Preformed non-HLA ETAR-Abs: >10 U/ml at baseline. Abs, antibodies; CsA, cyclosporine; eGFR, estimated glomerular filtration rate; ETAR, endothelin-1 type A receptor; EVR, everolimus; HLA, human leukocyte antigens; MPA, mycophenolic acid; TAC, tacrolimus.

Estimated glomerular filtration rate (eGFR) change from M1 to M12 was comparable in patients positive for performed non-HLA Abs vs. negative for non-HLA Abs (Table 4; Figures 1, 2). eGFR improvement was noted for EVR/TAC and MPA/TAC from baseline to Month 12, irrespective of non-HLA Abs positive status at baseline (AT1R-Abs: +3.2 ml/min/1.73 m^2^ and +10.1 ml/min/1.73 m^2^; ETAR-Abs: +5.0 ml/min/1.73 m^2^ and +7.0 ml/min/1.73 m^2^, respectively for EVR/TAC and MPA/TAC) or negative status at baseline (AT1R-Abs: +3.8 ml/min/1.73 m^2^ and +6.9 ml/min/1.73 m^2^; ETAR-Abs: +2.1 ml/min/1.73 m^2^ and +9.2 ml/min/1.73 m^2^). Patients who were on EVR/CsA with positive non-HLA Abs showed decrease in eGFR from M1 to M12 (AT1R-Abs: −2.7 ml/min/1.73 m^2^ and ETAR-Abs: −1.5 ml/min/1.73 m^2^; Figures 1, 2), whereas eGFR increased in patients that did not exhibit non-HLA Abs at baseline (AT1R-Abs: +5.9 ml/min/1.73 m^2^; ETAR-Abs: +5.4 ml/min/1.73 m^2^; Figures 1, 2).

## 4. Discussion

This exploratory ATHENA substudy was one of the first studies to compare the effect of EVR therapy with TAC or CsA vs. a MPA + TAC on *de novo* KTx patients regarding preformed non-HLA Abs, up to 12 months after transplantation.

Based on previous literature that preformed non-HLA Abs including Abs targeting AT1R and ETAR may have a very important role in affecting graft outcomes and survival after kidney transplantation, we were interested to look in a prospective trial with continuous immunosuppressive regimen exposure over 12 months and found that treatment with EVR or MPA with reduced CNI exposure was safe with no influence on graft outcomes. Results of biopsies in patients with a high level of these non-HLA Abs revealed featured characteristics of vascular acute or chronic injury (8–10). Banasik et al. conducted a study to evaluate the presence of non-HLA Abs at the time of transplant in 65 KTx recipients. The study results revealed that the high level of non-HLA Abs found in patients at the time of biopsy and graft loss in the non-HLA positive patients was also significantly higher (*p* = 0.00099) (10). In another study by Banasik et al., the presence of ETAR-Abs was evaluated in 116 KTx recipients, before and after transplantation. The study concluded that 47.4% of the analyzed KTx recipients were positive with ETAR-Abs before transplantation, and the presence of ETAR-Abs was linked to poor renal transplant function during the 1 year after transplantation (8).

Non-HLA Abs, specifically AT1R-Abs, are also responsible for activating the renin-angiotensin system. Studies have reported that AT1R-Abs have been linked to hypertension in pregnancy and malignant hypertension in non-transplant patients and are responsible for vascular pathology and severe hypertension in KTx recipients (31–34). Various treatment approaches, including valsartan, constitute an optimal therapy for the management of hypertension in KTx recipients. Dragun et al., examined losartan in KTx recipients with malignant hypertension and refractory rejection caused by AT1R-Abs. In comparison to individuals who did not take losartan, patients who did, demonstrated a much lower inflammatory infiltrate in the allograft biopsy and significantly higher graft survival (33).

It is considered that the primary target for non-HLA Abs is the vascular endothelium, which is responsible for hemodynamics regulation, angiogenic vascular remodeling, and metabolic, synthetic, and anti-inflammatory or antithrombogenic mechanisms (8). Various studies have reported that the significance of non-HLA Abs in KTx injury is unknown, although the impact of donor specific anti-HLA Abs is well understood (35–38). Banasik et al. reported that non-HLA Abs may participate in the arterial wall structural injury, which supports clothing and/or narrowing of arterial walls. Furthermore, non-HLA responses may be induced by cytokine-mediated endothelial cell activation (8). Additionally, AT1R-induced protein translation is mediated via the Akt-mTOR signaling pathway. Cellular metabolism, growth, proliferation, survival, and differentiation are all regulated by mTOR, specifically. It is the catalytic subunit of two mTOR signaling complexes, mTORC1 and mTORC2 (1). Recently, the ribosomal protein S6 kinase beta-1 (p-p70S6K) downstream effector of mTORC1 has been proposed as a diagnostic marker for antibody-mediated rejection in heart allografts (39). Various mTOR inhibitors including EVR are widely used in transplantation because of their antiproliferative effects (7, 40).

Our study is the first to definitively identify patients who were exclusively and only positive for preformed ETAR-Abs. From literature, different cut-offs have been used in the non-HLA Abs studies. Giral et al. and Lefaucheur C et al., considered the cut-off of AT1R-Abs at ≥10 U/ml and reported 47.2% and 20.1% of positivity in recipients, which was associated with high risk of developing acute rejection, antibody-mediated rejection and graft loss (2, 3). Taniguchi and Pearl MH et al. considered cut-off of AT1R-Abs at ≥15 U/ml and ≥17 U/ml, respectively, which was associated with allograft injury and graft failure (41, 42). Banasik et al. established the cut-off threshold of AT1R-Abs and ETAR-Abs of ≥9 U/ml and found higher risk of developing graft failure (9, 10). Moreover, in another study by Hiemann et al., in heart transplant recipients, the established cut-off threshold was ≥16 U/ml for the ETAR- and AT1R-Abs (12). In our study, we have set a cut-off threshold of >10 U/ml for both AT1R- and ETAR-Abs, and found 51% and 44% patients positive for ETAR-Abs and AT1R-Abs, respectively, at baseline.

Primary results of ATHENA study showed that EVR + reduced CNI is efficient, effective, and safe (good preservation of renal function and significant reduction of CMV and BKV infections in the EVR arm) (26). In addition to these results, the substudy presented here indicates that preformed non-HLA Abs have no correlation on clinical outcome. However, proportion of patients positive for non-HLA Abs from baseline to M12 reduced to a higher extent in EVR/TAC (AT1R-Abs: 42.9% to 17.7%; ETAR-Abs: 51.6% to 23.3%) compared to MPA/TAC (AT1R-Abs: 40.8% to 28.1%; ETAR-Abs: 46.7% to 34.7%) where it decreased from baseline to M6 but then increased slightly from M6 to M12. A possible explanation may be the specific ability of mTOR inhibitors to downregulate B-cells. In a comparative analysis of action of EVR and MPA on B-cell proliferation and differentiation into plasma cells, it was found that while MPA suppressed cell proliferation during initial phase of B-cell immune reaction, EVR could act in both early as well as later phase (43). A study showed that mTOR inhibitor reduced alloantibody production in transplant recipients via direct inhibition of alloprimed B cells while sparing the CD8^+^ antibody-suppressing T cells, and delayed graft rejection in both low and high alloantibody-producers (44). A recent study on molecular mechanism of action showed that non-HLA Abs targeting AT1R and ETAR induce endothelial injury via activation of PI3K/mTOR signaling, and the mTOR inhibitor, rapamycin abolish the activation of mTORC1 and mTORC2 after long term treatment with receptor antibodies. Thus, treatment with combination of mTOR inhibitors and receptor blockers may seem a future therapeutic approach in patients with non-HLA Abs-mediated allograft rejection (1). In line with the primary ATHENA study results (26), eGFR improvement was noted with MPA + TAC than in EVR groups. However, the number of patients was too low to draw any final conclusion. Liu et al. conducted a study in 79 recipients to evaluate the correlation between levels of AT1R- and ETAR-Abs and post-operative outcomes in KTx recipients. This study reported that the mean eGFR of patients was reduced significantly from 52.49 ± 24.96 to 42.58 ± 11.18 in the AT1R-Abs group and the AT1R- plus ETAR-Abs group showed a much lower eGFR (34.79 ± 15.27) (*p* = 0.008) (45). Various studies have reported that AT1R is an independent risk factor for graft loss and decline in eGFR (2, 8, 46). Hernández-Méndez et al. reported in a study of 111 patients that in KTx patients with AT1R-Abs, a lower median eGFR was observed compared to KTx patients with no Abs at 12 months (47). Our study also examined the patients solely positive for ETAR-Abs and evaluated the impact on clinical outcomes after transplantation. The findings showed that the ETAR-Abs were probably neutral, without any influence on the patients' clinical features.

The HLA antibodies may also play an important role in transplant rejection and patients with both HLA and non-HLA antibodies have been reported to have poor outcomes and lower graft survival (42). A separate substudy of ATHENA evaluated the effect of EVR vs. MPA in combination with reduced CNI on the formation of HLA antibodies and graft outcome in KTx patients (48). The results showed that incidence of *de novo* donor specific antibodies (DSA) was extremely low in all treatment groups. Similar to findings found for non-HLA, *de novo* DSA or preformed HLA antibodies had no influence on clinical outcomes in the first 12 months after KTx, irrespective of EVR or MPA exposure.

The main limitation of this study was that it was conducted for 12 months and there is lack of long-term data. The study was a post-hoc analysis and was not powered to detect statistically significant differences in clinical outcomes. A lack of differences between the groups may be due to small sample size. Other patient-related or immunological factors can contribute and additional research is needed to corroborate our findings and establish diagnostic or possibly targeted therapeutics.

In conclusion, given the existence of non-HLA Abs in up to 50% of patients and that preformed non-HLA Abs in all three immunosuppression regimens have no influence on clinical outcome, neither in terms of BPAR, AMR nor renal function, this study clearly demonstrated that the chosen combinations of immunosuppressants are safe and underscores the evidence of a minimal exposure of CNI for better patient management. However, further research on ETAR- and AT1R-Abs monitoring might be of interest in order to characterize immunological risk profiles in renal transplant recipients with high immunological risk and to identify potential immunologic events such as microvasculopathy and/or graft failure.

## Data Availability

Anonymized patient-level data from clinical trials may be shared by Novartis in a consortium called ClinicalStudyDataRequest.com (CSDR) in accordance with Novartis' policy for sharing clinical trial data. Requests to access the datasets should be directed to https://www.clinicalstudydatarequest.com/Study-Sponsors/Study-Sponsors-Novartis.aspx.
