# Supplementary material for: Impact of everolimus plus calcineurin inhibitor on formation of non-HLA antibodies and graft outcomes in kidney transplant recipients: 12-month results from the ATHENA substudy
Source: Front Transplant. 2023 Nov 21;2:1273890. doi: 10.3389/frtra.2023.1273890 (PMC11235374; doi:10.3389/frtra.2023.1273890)
Supplement: Supplementary file 1 [file Table1.docx]

Supplementary Material

**Impact of everolimus plus calcineurin inhibitor on formation of non-HLA antibodies and graft outcomes in kidney transplant recipients: 12-Month results from the ATHENA substudy**

**Aurélie Philippe*, Wolfgang Arns, Vanessa Ditt, Ingeborg A. Hauser, Friedrich Thaiss, Claudia Sommerer, Barbara Suwelack, Duska Dragun, Jan Hillen, Christiane Schiedel, Anja Elsässer, Björn Nashan***

***Corresponding author 1:** Aurélie Philippe

[aurelie.philippe@bih-charite.de](mailto:aurelie.philippe@bih-charite.de)

***Corresponding author 2:** Björn Nashan

[bjoern.nashan@gmail.com](mailto:bjoern.nashan@gmail.com)

# Supplementary Table

**Table S1.** **Demographic details for patients with non-HLA ETAR, AT1R, and both non-HLA AT1R and ETAR**

| *Patients positive with only non-HLA ETAR-Abs* | | | | | |
| --- | --- | --- | --- | --- | --- |
| **Variable** | **MPA+TAC (N=15) n (%)** | **EVR+TAC (N=12) n (%)** | **EVR+CsA (N=7) n (%)** | **Total (N=34) n (%)** | **p-value** |
|  | | | | | |
| **Age (yrs)** | | | | | |
| n; Mean (SD) | 15; 54.3 (13.5) | 12; 56.8 (8.4) | 7; 61.0 (7.2) | 34; 56.6 (10.8) | 0.406 * |
| **Age, n (%)** | | | | | |
| < 65 yrs | 13 (86.7) | 11 (91.7) | 6 (85.7) | 30 (88.2) |  |
| ≥ 65 yrs | 2 (13.3) | 1 (8.3) | 1 (14.3) | 4 (11.8) |  |
| **Gender, n (%)** | | | | | |
| Male | 12 (80.0) | 8 (66.7) | 4 (57.1) | 24 (70.6) | 0.626 ** |
| Female | 3 (20.0) | 4 (33.3) | 3 (42.9) | 10 (29.4) |  |
| **Race, n (%)** | | | | | |
| Caucasian | 15 (100.0) | 12 (100.0) | 7 (100.0) | 34 (100.0) |  |
| Non-Caucasian | 0 (0.0) | 0 (0.0) | 0 (0.0) | 0 (0.0) |  |
| *Patients positive with only non-HLA AT1R-Abs* | | | | | |
| **Variable** | **MPA+TAC (N=8) n (%)** | **EVR+TAC (N=4) n (%)** | **EVR+CsA (N=4) n (%)** | **Total (N=16) n (%)** | **p-value** |
|  | | | | | |
| **Age (yrs)** |  |  |  |  |  |
| n; Mean (SD) | 8; 53.5 (11.5) | 4; 49.5 (15.4) | 4; 47.3 (7.8) | 16; 50.9 (11.3) | 0.670 * |
| **Age, n (%)** | | | | | |
| <65 yrs | 7 (87.5) | 3 (75.0) | 4 (100.0) | 14 (87.5) |  |
| ≥65 yrs | 1 (12.5) | 1 (25.0) | 0 (0.0) | 2 (12.5) |  |
| **Gender, n (%)** | | | | | |
| Male | 5 (62.5) | 2 (50.0) | 1 (25.0) | 8 (50.0) | 0.804 ** |
| Female | 3 (37.5) | 2 (50.0) | 3 (75.0) | 8 (50.0) |  |
| **Race, n (%)** | | | | | |
| Caucasian | 8 (100.0) | 3 (75.0) | 4 (100.0) | 15 (93.8) | 0.500 ** |
| Non-Caucasian | 0 (0.0) | 1 (25.0) | 0 (0.0) | 1 (6.3) |  |
| *Patients with both positive non-HLA AT1R- and ETAR-Abs* | | | | | |
| **Variable** | **MPA+TAC (N=41) n (%)** | **EVR+TAC (N=35) n (%)** | **EVR+CsA (N=26) n (%)** | **Total (N=102) n (%)** | **p-value** |
| **Age (yrs)** | | | | | |
| n; Mean (SD) | 41; 56.8 (11.5) | 35; 52.1 (12.2) | 26; 54.8 (12.1) | 102; 54.7 (12.0) | 0.235 * |
| **Age, n (%)** | | | | | |
| < 65 yrs | 31 (75.6) | 29 (82.9) | 19 (73.1) | 79 (77.5) |  |
| ≥ 65 yrs | 10 (24.4) | 6 (17.1) | 7 (26.9) | 23 (22.5) |  |
| **Gender, n (%)** | | | | | |
| Male | 29 (70.7) | 24 (68.6) | 19 (73.1) | 72 (70.6) | 0.962 ** |
| Female | 12 (29.3) | 11 (31.4) | 7 (26.9) | 30 (29.4) |  |
| **Race, n (%)** | | | | | |
| Caucasian | 41 (100.0) | 32 (91.4) | 26 (100.0) | 99 (97.1) | 0.053 ** |
| Non-Caucasian | 0 (0.0) | 3 (8.6) | 0 (0.0) | 3 (2.9) |  |

*F-test; **Fisher's exact test. Preformed non-HLA AT1R-Abs: >10 U/mL at baseline; preformed non-HLA ETAR-Abs: >10 U/ml at baseline.

Abs, antibodies; AT1R, angiotensin II type 1 receptor; CsA, cyclosporine A; EVR, everolimus; ETAR, endothelin-1 type A receptor; HLA, human leukocyte antigen; MPA, mycophenolic acid; SD, standard deviation; TAC, tacrolimus; yrs, years
